# CD160 Promotes NK Cell Functions by Upregulating Glucose Metabolism and Negatively Correlates With HIV Disease Progression

**DOI:** 10.3389/fimmu.2022.854432

**Published:** 2022-08-19

**Authors:** Zheng Sun, Yidi Li, Zining Zhang, Yajing Fu, Xiaoxu Han, Qinghai Hu, Haibo Ding, Hong Shang, Yongjun Jiang

**Affiliations:** ^1^ National Health Commission (NHC) Key Laboratory of Acquired Immunodeficiency Syndrome (AIDS) Immunology (China Medical University), National Clinical Research Center for Laboratory Medicine, The First Affiliated Hospital of China Medical University, Shenyang, China; ^2^ Key Laboratory of Acquired Immunodeficiency Syndrome (AIDS) Immunology, Chinese Academy of Medical Sciences, Shenyang, China; ^3^ Key Laboratory of Acquired Immunodeficiency Syndrome (AIDS) Immunology of Liaoning Province, Shenyang, China; ^4^ Collaborative Innovation Center for Diagnosis and Treatment of Infectious Diseases, Hangzhou, China

**Keywords:** CD160, NK cells, glucose metabolism, TGF-β1, PI3K/AKT/mTOR/s6k signaling pathway, HIV

## Abstract

Natural killer (NK) cells are crucial for immune responses to viral infections. CD160 is an important NK cell activating receptor, with unknown function in HIV infection. Here, we found that CD160 expression was reduced on NK cells from HIV-infected individuals and its expression was negatively correlated with HIV disease progression. Further, GLUT1 expression and glucose uptake were higher in CD160^+^ NK cells, and the results of RNA-seq and flow cytometry demonstrated that CD160 positively regulated glucose metabolism through the PI3K/AKT/mTOR/s6k signaling pathway, thereby enhancing NK cell function. Moreover, we determined that reduced CD160 expression on NK cells could be attributed to the higher plasma levels of TGF-β1 in HIV-infected individuals. Overall, these results highlight the vital role of CD160 in HIV disease progression and regulation of glucose metabolism, indicating a potential target for HIV immunotherapy.

## Introduction

Natural Killer (NK) cells are cytotoxic effector lymphocytes with a crucial role in innate immune responses to viral infections (1). Human NK cells account for 10%–15% of all peripheral blood lymphocytes and are phenotypically distinguished by the presence of surface CD56 and/or CD16 on the surface and the lack of CD3 expression (2). NK cell functions are regulated by a considerable number of co-signaling receptors, and changes in the balance between receptors that mediate inhibitory or activating signals determine the degree of NK cell activation (1, 3). Due to increased stimulation of activating receptors and/or a lack of signals through inhibitory receptors, the balance shifts toward NK cell activation; however, the factors that regulate the balance between inhibitory and activating stimuli are poorly understood (4). During early viral infection, NK cells express higher surface levels of activating receptors, and can efficiently kill virus-infected cells (5). Viral infection progression to a chronic state is associated with reduced expression of activating receptors, resulting in specific defects in activating receptor-mediated NK cell activation (6). Hence, activating receptors are considered important for viral control (7). Cellular metabolism is now also considered a significant factor that can determine NK cell fate and function, and recent evidence suggests that NK cells are primarily fueled by glucose (8). For example, IFN production can be metabolically controlled at several levels, including during transcription, translation, or post-translational processing. Glucose can be used by NK cells to generate ATP and NADPH *via* several metabolic pathways or as a carbon source for other macromolecules, such as amino acids and fatty acids (9). Therefore, continued study of NK cell glucose metabolism is vital to determine how NK cells can be modified to withstand viral infection or tumor cells.

Human CD160 (also known as BY55) is 181 amino acid protein, which should give a total mass of 19.81 kDa (10) that was initially discovered by Bensussan A and co-workers in 1993 (11). The human *CD160* gene is located on chromosome 1 (12) and CD160 protein is a transmembrane glycoprotein that is glycosylphosphatidylinositol-anchored and belongs to the immunoglobulin superfamily (IgSF) (12). CD160 is found on a variety of immune cells, including NK cells, a small fraction of CD4^+^ T cells and CD8^+^ T cells, and intestinal intraepithelial T lymphocytes, and CD160 is also present in CD4^+^ T cells within inflammatory skin infiltrates (13) and in activated endothelial cells (12, 14, 15).

CD160 preferentially binds to the herpes virus entry mediator (HVEM), and engagement of CD160 with this ligand activates NK-cell function, causing increased IFN-γ and TNF-α secretion, and in the tumor microenvironment, the activation of CD160 on immune cells promotes target cell cytolysis (16). In addition, the cytotoxic function mediated by CD160 is independent of the crosslinking of other activating receptors, hence, CD160 functions as a distinct activating receptor capable of increasing cytokine production in response to specific ligation. Other NK cell activating receptors, such as NKG2D are unable to trigger by themselves IFN-γ production in humans (17, 18). Thus CD160 differs from several other activating NK cell receptors, suggesting a crucial role for CD160 in NK-cell cytokine production and cytotoxicity. Furthermore, an investigation of patients with hepatocellular carcinoma revealed that reduced CD160 result in impaired NK-cell function and poor clinical prognosis (19). Moreover, data from CD160(-/-) mice showed that CD160 is essential for IFN-γ production by NK cells (20). These results suggest that CD160 is critical to NK cell immune function, while the potential mechanism by which CD160 regulates NK cell function has not been studied in depth.

Human immunodeficiency virus (HIV) infection is featured by profound innate and adaptive immune system dysfunction, resulting in tumors or opportunistic infections (21–23). HIV infection can induce NK cell functional exhaustion, which manifests as impairments in cytokine production, cytotoxic function, and antibody-dependent cell-mediated cytotoxicity (24). The repertoire and function of NK cell receptors are altered during HIV-1 infection; for instance, treatment-naive HIV-infected individuals exhibit substantial decrease in CCR7^+^ CD56^bright^ NK cells, which are associated with higher viral loads (25). Nevertheless, little is known about CD160 expression on human NK cells and determining the mechanism underlying the contribution of CD160 to NK cell activity against HIV infection has potential to reveal novel strategies for antiretroviral therapy (ART).

In this study, we identified altered CD160 expression on NK cells from HIV-infected individuals and its association with disease progression. Next, we explored the effect of CD160 on NK cell function and the underlying metabolic pattern. To further characterize peripheral CD160^+^ NK cells, we analyzed related signaling pathways in HIV-infected individuals, and transcriptomes from CD160^+^ and CD160^-^ NK cells, to elucidate the roles of CD160 in HIV infection. Further, we explored the mechanism responsible for altered CD160 expression. Co-signaling receptors have become increasingly attractive treatment targets, due to success in immunotherapy (26). This research provides new insights into immunotherapy for HIV infection.

## Materials and Methods

### Study Participants

In total, 174 HIV-infected individuals were recruited from the First Affiliated Hospital of China Medical University, and 77 HIV-negative controls with no immune system disorders and normal routine blood examination. Individuals infected with HIV were all male, and their median age was 34 years (range, 17–66 years). Among HIV-negative controls, 95% were male and their median age was 38 years (range, 19–69 years) (**Table 1**). The Medical Research Ethics Committee of the First Affiliated Hospital of China Medical University approve for this investigation, and written informed consents for participation were provided by all study participants.

**Table 1:** Characteristics of clinical study participants.

Study group	HIV (n = 174)	NC (n = 77)	P-value*
Age, mean (range), years	34 (17 — 66)	38 (19 — 69)	0.3198
Sex, no. (%)	Male: 174 (100)	Male: 73 (95) Female: 4 (5)	
HIV status	HIV^+^	HIV^-^	
Treatment status, no.	ART naïve: 121 ART: 53	**—**	
Median CD4+ T cell counts (range), cells/mm³	395 (1 — 1233)	841 (478 —1 375)	**<0.0001**
Plasma level of HIV RNA (copies/mL)	128640 (< 20 — 2790000)	NA	**—**

*HIV^+^ vs HC. ART, antiretroviral therapy; NA, not applicable; Boldface p values indicate p < 0.05.

### Phenotypic Analysis

The phenotype of freshly isolated PBMCs were analyzed by flow cytometry. The following fluorochrome-conjugated mAbs and corresponding isotype control antibodies were used: APC-cy7-CD3 (clone: UCHT1; Biolegend), Percp-cy5.5-CD14 (clone: M5E2; Biolegend), Percp-cy5.5-CD19 (clone: HIB19; Biolegend), APC-CD16 (clone: 3G8; Biolegend), PE-cy7-CD56 (clone: HCD56; Biolegend), PE-CD160 (clone: BY55; Biolegend), PE-IgM (clone: MM-30; Biolegend), Brilliant Violet 421-CD69 (clone: FN50; Biolegend), Brilliant Violet 421-IgG1 (clone: MOPC-21; Biolegend), Alexa Fluor^®^ 488-GLUT1 (clone: 202915; R&D Systems), PE-CD36 (clone: CB38; BD PharMingen™), PE-IgM (clone: G155-228; BD PharMingen™), APC-CD71 (clone: CY1G4; Biolegend), APC-IgG2a (clone: MOPC-173; Biolegend), Brilliant Violet 510-CD98 (clone: UM7F8; BD OptiBuil™), and Brilliant Violet 510-IgG1 (clone: X40; BD OptiBuil™). Fixable Viability Stain 620 (BD Horizon™, USA) was used to evaluate cell viability in all experiments. The phenotypic analysis above was conducted using a BD FACS Canto II Flow Cytometer and analyzed by FlowJo 10.4 software (Ashland, OR, USA).

### Analysis of NK Cell Glucose Uptake Capacity

The fluorescent glucose analog, 2-NBDG (Invitrogen), was used to measure NK cell glucose uptake. Isolated cells were resuspended in glucose-free medium (Life Technologies) that had been prewarmed (37°C), 3.4 µL of the dissolved 2-NBDG solution added, incubated at 37°C for 30 min and washed with PBS (300 g, 10 min). Before detection by flow cytometry, cells were labeled with surface markers (CD3, CD14, CD19, CD16, CD56, and CD160).

### NK Cell Function Assays

For NK-cell activation assays, PBMCs from HIV-infected individuals were seeded in 96-well U bottom plates and stimulated with a cytokine cocktail (IL-12, IL-15, and IL-18 at 10, 50, and 100 ng/ml, respectively; R&D Systems, USA). Unstimulated cells were used as negative controls. Cells were incubated for 24 h at 37°C with 5% CO_2_ in RPMI medium, supplemented with 10% FBS. During the final 4 h of culture, GolgiStop (1 µL; BD Biosciences, USA) was added. Cells were stained with APC-cy7-CD3 (clone: UCHT1; Biolegend), Percp-cy5.5-CD14 (clone: M5E2; Biolegend), Percp-cy5.5-CD19 (clone: HIB19; Biolegend), APC-CD16 (clone: 3G8; Biolegend), PE-cy7-CD56 (clone: HCD56; Biolegend), PE-CD160 (clone: BY55; Biolegend) on ice protect from light for 20 min. Cells were then fixed for 20 min at 4°C in the dark with Fixation/Permeabilization solution (BD Biosciences), then washed twice with Perm/Wash buffer (BD Biosciences), and intracellularly stained with IFN-γ-Alexa Fluor^®^ 488 (clone: 4S.B3; Biolegend), TNF-α-BV421 (clone: MAb1; BioLegend) for 20 min, and analyzed using a BD FACS Canto II Flow Cytometer (BD Biosciences).

For the NK cell proliferation assay, PBMCs were stimulated and incubated as described above. After staining with surface markers, then fixed for 45 min at 4°C in the dark with 1 ml Fixation/Permeabilization working solution (eBioscience™, USA), washed twice with Permeabilization Buffer (eBioscience™, USA), and Brilliant Violet 421 anti-human Ki-67 (clone: Ki-67; BioLegend) antibody used to stain intracellular Ki67.

For the CD160-activation assay, cells were seeded in presence of 10 µg/ml of soluble CL1-R2 antibody (clone: CL1-R2; MBL International), an agonist antibody for CD160 (27, 28), or the corresponding mIgG control (clone: MOPC-21; BioLegend) for 1 h, then stimulated with IL-12, IL-15, and IL-18 for 24 h at 37°C with 5% CO_2_, followed by evaluation of IFN-γ release as described above.

To detect the effect of exogenous glucose or 2-DG on NK cell function, PBMCs were seeded in 96-well U bottom plates in RPMI medium, supplemented with 10% FBS. A cytokine combination (IL-12, IL-15 and IL-18) was used to activate the cells; unstimulated cells served as a negative control. Then, 10 mmol/L glucose or 5mmol/L 2-DG was added to the wells, cell culture, surface labeling, and intracellular IFN-γ staining were then performed as described above.

To detect GLUT1 expression under the influence of exogenous glucose or CD160 activation, PBMCs from HIV-infected individuals were resuspended and stimulated as described above, then added 10 mmol/L glucose, 10 µg/ml CL1-R2 antibody (clone: CL1-R2; MBL International), or corresponding mIgG control (clone: MOPC-21; BioLegend) to the wells, and GLUT1 expression on NK cells was measured by flow cytometry.

### Detection of Phosphorylation Levels

Negative selection was used to extract purified NK cells from whole blood (Stemcell). Cells were incubated in medium alone (used to define gates) or with mIgG control (BioLegend, USA) or CL1-R2 antibody (MBL International) for 30 min at 37^°^ C with 5% CO2, and then treated with BD Phosflow™ Perm Buffer III and BD Phosflow™ Fix Buffer I following the manufacturer’s protocol. PE-mTOR (clone: O21-404; BD Phosflow), Brilliant Violet 421-AKT (clone: M89-61; BD Phosflow), or Alexa Fluor^®^ 488-s6k (clone: 215247; R&D Systems) were added to cells combined with mIgG control (BioLegend, USA) or CL1R2 antibody (MBL International). Phosphorylation of mTOR, AKT and s6k was measured by flow cytometry.

### GSEA

RNA was extracted from freshly isolated peripheral CD160^-^ and CD160^+^ NK cells using TRIzol reagent (Invitrogen), then sequenced on the BGISEQ-500 platform (BGI, Shenzhen, Guangdong, China). GSEA v3.0 software (Broad Institute software) was used for transcriptome GSEA. Enrichment scores determined by GSEA were used to compare different KEGG pathways between CD160^-^ and CD160^+^ NK cells. The following thresholds satisfied simultaneously, were considered meaningful: |NES|>1, Nominal p-value<0.05, FDR q-value<0.25.

### ELISA

Plasma was collected from ART-naïve HIV-infected individuals and negative controls, and stored at -80°C. TGF-β1 was assessed using a TGF-β1 Human ELISA Kit (Invitrogen, USA) according to the manufacturer’s instructions.

### Detection of CD160 Expression Under the Influence of TGF-β1

CD160 expression on NK cells was evaluated using flow cytometry after PBMCs were cultured in medium alone or with recombinant TGF-β1 (10 ng/ml; PeproTech, USA) for 24 hours.

### Statistical Analysis

The non-parametric Mann-Whitney U test and non-parametric Wilcoxon matched-pairs signed-rank test were used for evaluation of differences in data between two groups. The Spearman’s rank test was used for analysis of correlation between two groups. Values of *p <* 0.05 were considered statistically significant. All data analysis were performed using Prism Version 9.0 (GraphPad Software, USA) software.

## Results

### Expression of CD160 on NK Cells Is Reduced in HIV-Infected Individuals and Negatively Correlates With HIV Disease Progression

**Figure 1:**
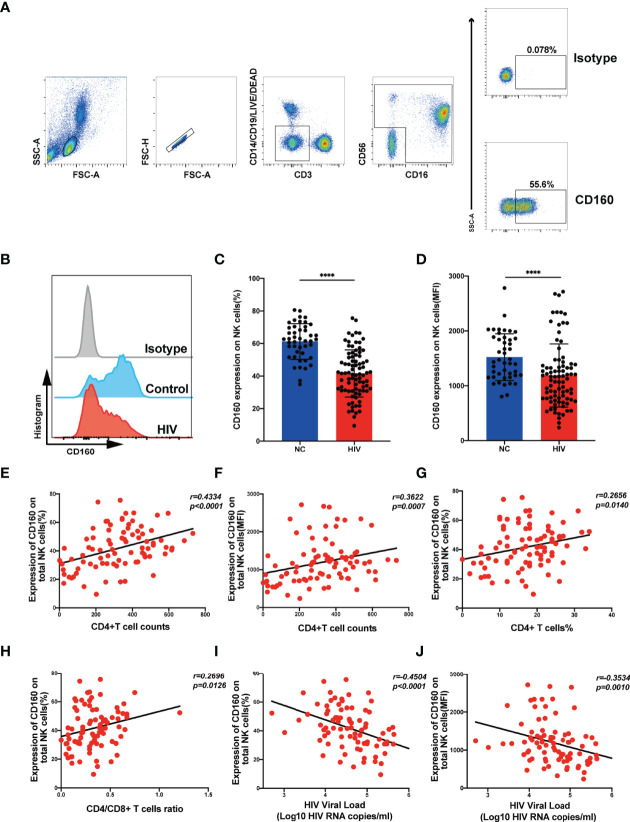
CD160 expression on NK cells is reduced in HIV-infected individuals and negatively associated with HIV disease progression. **(A)** Gating strategy for human CD160^+^ NK cells. Peripheral blood mononuclear cells were stained with Fixable Viability Stain 620 (Percp-cy5.5), anti-CD3 (APC-cy7), anti-CD14 (Percp-cy5.5), anti-CD19 (Percp-cy5.5), anti-CD16 (APC), anti-CD56(PE-cy7), and anti-CD160 (PE) monoclonal antibodies, and total NK cells were determined as CD3/CD14/CD19-negative lymphocytes expressing CD56 and/or CD16. CD160 expression was gated based on an isotype control. **(B)** Representative histograms showing phenotypic results for HIV-infected individuals and HIV-negative controls (NC). Light-gray, Isotype control; blue, HIV-negative controls; red, HIV-infected individual. **(C, D)** Percentage **(C)** and mean fluorescence intensity (MFI) **(D)** of CD160 expression on total NK cells in HIV-infected individuals (n = 85) and negative controls (n = 45). **(E, F)** Correlation between the percentage **(E)** or MFI **(F)** of CD160 expression on total NK cells and CD4^+^ T cell counts (cells/mm^3^) in HIV-infected individuals (n = 85). **(G)** Correlation between the percentage of CD160^+^ NK cells and the proportion of CD4^+^ T cells in peripheral blood from HIV-infected individuals (n = 85). **(H)** Correlation between the percentage of CD160 expression on total NK cells and CD4/CD8 T cell ratio in peripheral blood from HIV-infected individuals (n = 85). **(I, J)** Correlation between the percentage **(I)** or MFI **(J)** of CD160 expression on total NK cells and HIV plasma viral load (VL) in HIV-infected individuals (n = 84). The mean fluorescence intensity of CD160 in panels **(D, F, J)** was from total NK cells. A non-parametric Mann-Whitney U test was used for comparisons between two groups. A Spearman’s rank test was applied for correlation analysis; ****P < 0.0001; ns, no significance. In **(C, D)**, data are represented as means ± SD.

To investigate the expression of CD160 on peripheral total NK cells, samples from HIV-negative control and HIV groups were analyzed by flow cytometry (**Figures 1A, B** **)**, CD160 expression levels on total NK cells were significantly lower in the HIV group than NC group (**Figure 1C**), with mean fluorescent intensity values also relatively reduced in the HIV group (**Figure 1D**), and ART treatment cannot significantly increase CD160 expression on total NK cells (**Figures S3A, B** **)**. Based on the expression of CD56 and CD16, NK cells were divided into three major subsets (CD56^bright^, CD56^dim^, and CD56^-^CD16^+^) for further analysis **(** **Figure S1A**). CD160 on NK-cell subsets from the HIV group were lower than those from the NC group (**Figures S1B–D**). Further, mean fluorescent intensity of CD160 in CD56^bright^ and CD56^dim^ NK cells was lower in the HIV group, whereas no significant difference existed between the two groups in CD56^-^CD16^+^ NK cells (**Figures S1E–G**).

Next, we evaluated the association of CD160 expression on total NK cells with CD4^+^ T cell counts and plasma viral load, which are considered significant clinical indicators of HIV disease progression. The frequency of CD160-positivity on total NK cells was positively correlated with CD4^+^ T cell counts (**Figure 1E**), as was CD160-positivity on CD56^dim^ NK and CD56^-^CD16^+^ NK subsets (**Figures S2C, E**). Similarly, CD160 mean fluorescent intensity values on total NK cells and the CD56^dim^ and CD56^-^CD16^+^ NK subsets were positively correlated with CD4^+^ T cell counts (**Figures 1F**, **S2D, F**). Further, plasma viral load was negatively correlated with CD160-positivity on total NK cells and the same two NK cell subsets (**Figures 1I**, **S2I, K**), with consistent findings for mean fluorescent intensity of CD160 (**Figures 1J**, **S2J, L**). In contrast, the frequencies and mean fluorescent intensity values of CD160 on CD56^bright^ NK cells was correlated with neither CD4^+^ T cell counts nor plasma viral load (**Figures S2A, B, G, H**). In addition, the frequency of CD160-positivity on total NK cells was positively correlated with the percentage of CD4^+^T cells (**Figure 1G**) and the CD4^+^/CD8^+^T cell ratio (**Figure 1H**). These results suggest that the expression of CD160 on peripheral total NK cells from HIV-infected individuals may play a vital role in disease progression.

### CD160^+^ NK Cells Have Higher Functions and Activation of CD160 Molecules Promotes NK Cell Functions

**Figure 2:**
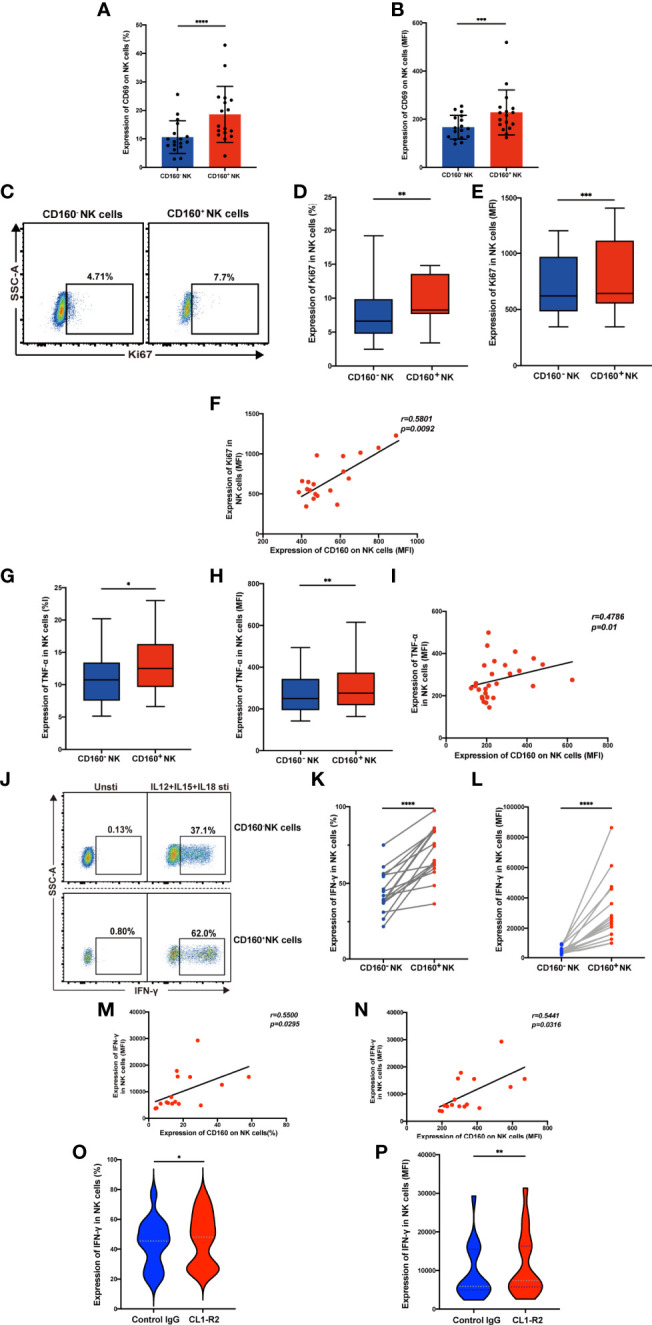
Expression of CD160 enhances NK cell effector function and activating CD160 improves IFN-γ production. **(A, B)** Comparison the expression of CD69, the NK cell activation marker, between CD160^-^ and CD160^+^ NK cells in HIV-infected individuals (n = 17). PBMCs from HIV-infected individuals were stimulated with IL-12 + IL-15 + IL-18 (10, 50 and 100 ng/ml, respectively) for 24 h to detect the following NK cell functions **(C–N)**. **(C)** Ki67 expression in CD160^-^ and CD160^+^ NK cells measured by flow cytometry. Plots are representative of data from HIV-infected individuals. **(D, E)** Paired comparisons of percentage **(D)** and MFI of Ki67 expression **(E)** between CD160^–^ and CD160^+^ NK cells in HIV-infected individuals (n = 19). **(F)** Correlation between the MFI value of CD160 and Ki67 expression in total NK cells from HIV-infected individuals (n = 19). **(G, H)** Percentages **(G)** and MFI of TNF-α **(H)** expression in CD160^-^ and CD160^+^ NK cells from HIV-infected individuals (n = 28). **(I)** Correlation between the MFI of CD160 expression and TNF-α production in total NK cells from HIV-infected individuals (n = 28). **(J)** Representative cytometry dot plots showing percentages of IFN-γ-producing CD160^-^ and CD160^+^ NK cells in HIV-infected individuals. **(K, L)** Paired comparison of the percentages **(K)** or MFI **(L)** of IFN-γ production between CD160^-^ and CD160^+^ NK cells in HIV-infected individuals (n = 16). **(M, N)** Correlation between the MFI **(M)** or percentage **(N)** of CD160 expression on total NK cells and the MFI of IFN-γ expression in total NK cells (n = 16). **(O, P)** PBMCs were treated with 10 µg/ml CL1-R2 antibody (anti-CD160 agonistic mAb) or negative control IgG1 for 1h and then stimulated with IL-12 + IL-15 + IL-18 (10, 50 and 100 ng/ml, respectively) for 24 h, then percentages **(O)**, MFI **(P)** of IFN-γ production in total NK cells from HIV-infected individuals were analyzed (n = 16). The mean fluorescence intensity of CD69, Ki67, TNF-α, and IFN-γ in **(B, E, H, L)** was from CD160^-^ and CD160^+^ NK cells, respectively. And the mean fluorescence intensity of CD160, Ki67, TNF-α, and IFN-γ in **(F, I, M, N, P)** was from total NK cells. A Wilcoxon matched-pairs signed-rank test was employed for paired-group comparisons. A Spearman’s rank test was applied for correlation analysis; * P < 0.05; ** P < 0.01; *** P < 0.001; **** P < 0.0001. Data are represented as means ± SD.

CD160 plays a crucial role in regulating NK cell function in healthy people (15). Here, we compared CD160^+^ and CD160^-^ NK cell function in HIV-infected individuals. Both the frequencies and the mean fluorescent intensity of the NK cell activation marker, CD69, were higher in CD160^+^ NK cells than CD160^-^ NK cells in HIV-infected individuals (**Figures 2A, B** **)**, indicating that increased CD160 expression is associated with higher NK cell activity. We also detected the expression of CD16 on both CD160^-^ and CD160^+^ NK cells, and found the expression level of CD16 on CD160^+^ NK cells was significantly higher than that on CD160^-^ NK cells in HIV-infected individuals, indicating that CD160^+^ NK cells might possibly have a stronger ADCC effect in HIV-infected individuals (**Figures S4A, B** **)**.

CD160 acts as an activating receptor on total NK cells; however, there is little information available about the relationship between CD160 and NK-cell proliferative capacity. We found that Ki67 expression was higher in CD160^+^ NK cells (**Figures 2C-E**), Ki67 expression in total NK cells positively correlated with CD160 expression on total NK cells in HIV-infected individuals with cytokine stimulation (**Figure 2F**), suggesting that CD160 plays a critical role in NK-cell proliferation in HIV-infected individuals. Then we examined the effect of CD160 expression on NK cell cytokine production in patients infected with HIV. Intracellular NK cell staining assays revealed that TNF-α (**Figures 2G, H** **)** and IFN-γ (**Figures 2J-L**) expression levels were significantly higher in cytokine-activated CD160^+^ NK cells from HIV-infected individuals, and that, both were positively correlated with CD160 expression on total NK cells from HIV-infected individuals (**Figures 2I, M, N** **)**. We used CL1-R2, an anti-CD160 antibody, to activate CD160 signaling, followed by cytokine stimulation, to identify whether CD160 is directly involved in controlling NK cell function (29). As expected, we observed that CL1-R2 significantly enhanced IFN-γ production in total NK cells from HIV-infected individuals (**Figures 2O, P** **)**. While CD107a expression was comparable between CD160^-^ and CD160^+^ NK subsets from HIV-infected individuals on cytokine stimulation or cocultured with K562 cells (data not shown). In summary, these data indicated that CD160 expression defines a proliferative and functional NK-cell subset.

### CD160 Expression Is Associated With Enhanced Glucose Transport and Uptake Capacity of NK Cells

**Figure 3:**
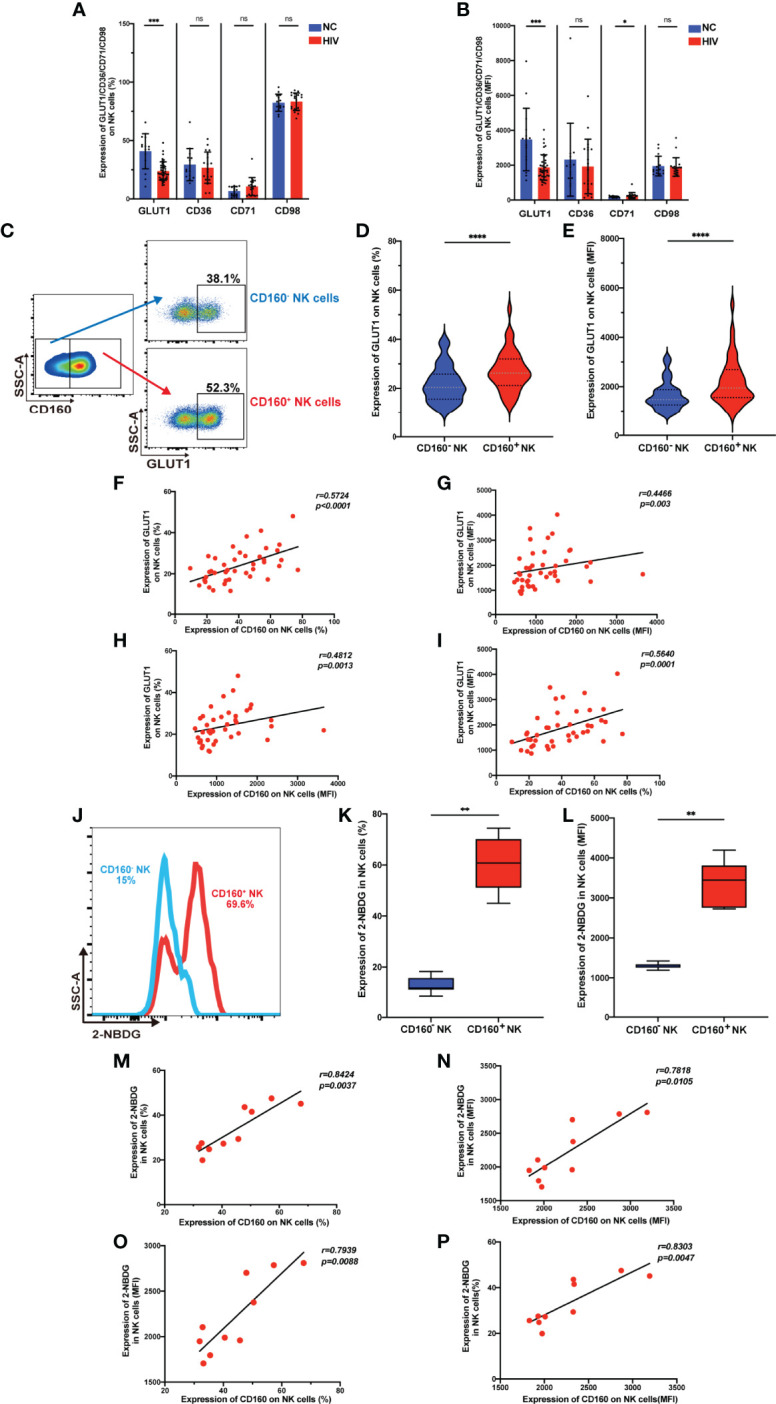
CD160 expression is associated with enhanced glucose transport and uptake capacity of NK cells. **(A, B)** Percentages **(A)** and MFI **(B)** of GLUT1 (GLUT1: NC, n = 15; HIV, n = 43), CD36, CD71, CD98 (CD36, CD71, CD98: NC, n = 14; HIV, n = 19) on total NK cells from HIV-infected individuals were determined by flow cytometric analysis. **(C)** Representative cytometry dot plots for GLUT1 expression on CD160^-^ and CD160^+^ NK cells in HIV-infected individuals. **(D, E)** Comparison of percentages **(D)** and MFI **(E)** of GLUT1 expression between CD160^–^ and CD160^+^ NK cells in HIV-infected individuals (n = 42). **(F–I)** Correlation between CD160 and GLUT1 expression on total NK cells (n = 42). **(J)** Representative histograms showing 2-NBDG uptake by CD160^-^ and CD160^+^ NK cells in HIV-infected individuals. **(K, L)** Paired comparison of the percentages **(K)** and MFI **(L)** showing 2-NBDG uptake between CD160^–^ and CD160^+^ NK cells in HIV-infected individuals (n = 10). **(M–P)** Correlation between CD160 expression and 2-NBDG uptake in total NK cells from HIV-infected individuals (n = 10). The mean fluorescence intensity of GLUT1, CD36, CD71, CD98, and CD160 in **(B, G, H, I, N, O, P)** was from total NK cells. And the mean fluorescence intensity of GLUT1 and 2-NBDG in **(E, L)** was from CD160^-^ and CD160^+^ NK cells, respectively. A non-parametric Mann-Whitney U test was used for comparisons between two groups. A Wilcoxon matched-pairs signed-rank test was applied for paired-group comparisons. A Spearman’s rank test was used for analysis of correlation; * P < 0.05; ** P < 0.01; *** P < 0.001; **** P < 0.0001; ns, no significance. Data are represented as means ± SD.

It is established that metabolism is essential for maintaining the development and function of NK cells (30), however, no association of the CD160 protein with metabolism has been reported previously. We examined the expression of metabolism dedicated transporters on total NK cells from the HIV and NC groups, and found down-regulation of GLUT1 in HIV-infected individuals (**Figures 3A, B** **)**, while there were no differences in the frequencies of the fatty acid transporter, CD36, the transferrin receptor, CD71, or the acid transporter, CD98. Next, we measured the expression of GLUT1 on CD160^-^ and CD160^+^ NK cells and found the percentage and mean fluorescent intensity of GLUT1 were increased in CD160^+^ NK cells from HIV-infected individuals (**Figures 3C–E**), similar results were found in NC group (**Figures S5A, B** **)**. Moreover, the expression of CD160 and GLUT1 were found to be positively correlated on total NK cells in HIV-infected individuals (**Figures 3F-I**). Furthermore, flow cytometric analysis showed that CD160^+^ NK cells from HIV-infected individuals exhibited enhanced uptake of the metabolic substrate, 2-(N-(7-nitro-benz-2-oxa-1,3-diazol-4-yl) amino)-2-deoxyglucose (2-NBDG; a fluorescent glucose analog) (31), relative to CD160^-^ NK cells (**Figures 3J–L**). Further, the percentage and mean fluorescence intensity of CD160 and 2-NBDG were correlated closely with one another (**Figures 3M–P**). Together, these findings demonstrate, for the first time, that there is an association between CD160 expressing NK cells and glucose uptake in HIV-infected individuals.

### CD160^+^ NK Cells From HIV-Infected Individuals Are Significantly Influenced by Glucose Addition or Inhibition of Glycolysis

Glucose is the major cellular fuel that promotes NK-cell survival and effector functions (32), and our data showed that there is an association between CD160 expressing NK cells and glucose uptake in HIV-infected individuals. Therefore, we hypothesized that additional glucose could rescue the functional defects mediated by lack of CD160 in NK cells during HIV infection. Therefore, we investigated whether addition of exogenous glucose could improve CD160-associated NK-cell activation.

**Figure 4:**
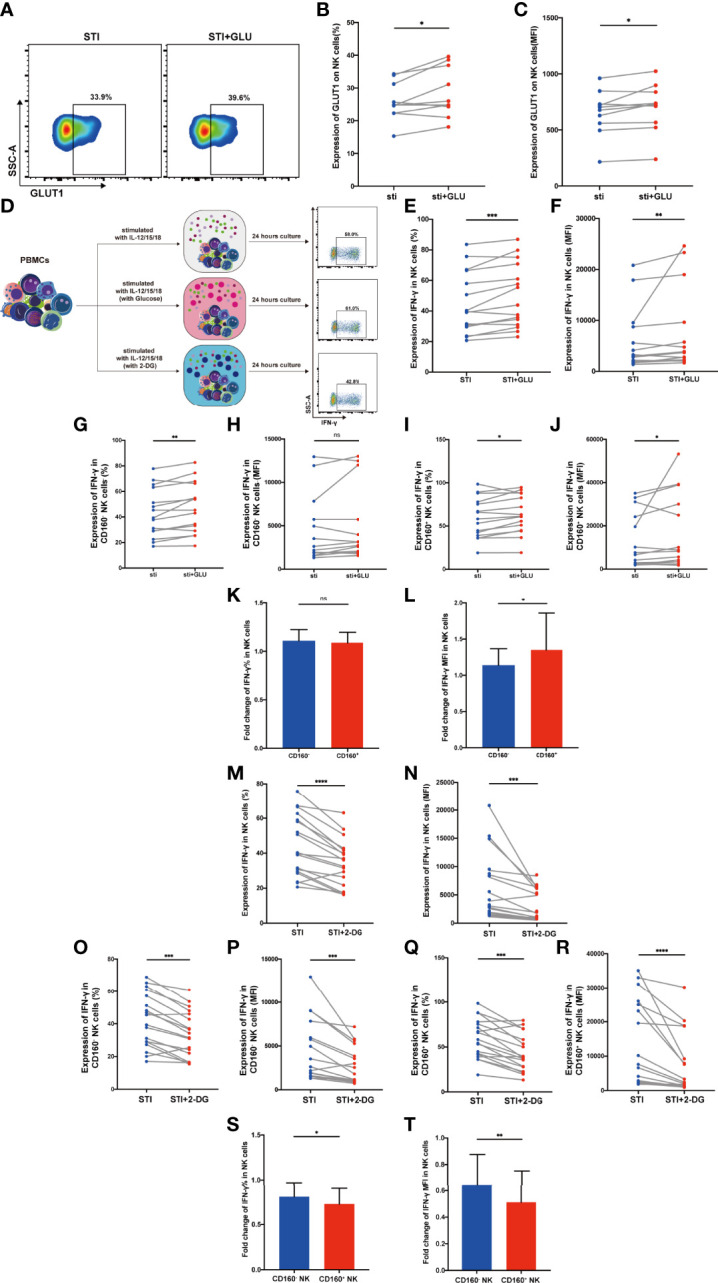
CD160^+^ NK cells from HIV-infected Individuals were significantly influenced by addition of glucose or glycolysis inhibition. **(A)** Representative dot plots of GLUT1 expression on total NK cells from HIV-infected individuals was measured by flow cytometry after 24 h of cytokines stimulation (10 ng/ml IL-12 + 50 ng/ml IL-15 + 100 ng/ml IL-18) with or without additional 10 mmol/L glucose. Corresponding statistical analysis of the percentage **(B)** and MFI **(C)** of GLUT1 on NK cells (n = 10). **(D)** Schematic diagram of experiments using PBMCs treated with IL-12 + IL-15 + IL-18 (10, 50 and 100 ng/ml, respectively) alone, additional glucose (10 mmol/L) or 2-DG (5 mmol/L) to study effects on IFN-γ production in total NK cells from HIV-infected individuals. **(E, F)** Corresponding statistical analysis of the percentage **(E)** and MFI **(F)** of IFN-γ in total NK cells from HIV-infected individuals with or without additional glucose (10 mmol/L) (n = 15). **(G–J)** Paired comparison of the percentage **(G, I)** and MFI **(H, J)** of IFN-γ in CD160^-^ or CD160^+^ NK cells from HIV-infected individuals with or without additional glucose (10 mmol/L) (n = 15). **(K, L)** Fold changes of the percentage **(K)** and MFI **(L)** of IFN-γ expression were calculated as the treatment with additional glucose (10 mmol/L) over the treatment without additional glucose in CD160^-^ as well as CD160^+^ NK cells from HIV-infected individuals (n = 15). **(M, N)** Percentage **(M)** and MFI **(N)** of IFN-γ expression in total NK cells with or without 2-DG treatment (5 mmol/L) (n = 18). **(O–R)** Paired comparisons of the percentage **(O, Q)** and MFI **(P, R)** of IFN-γ in CD160^-^ or CD160^+^ NK cells with or without additional 2-DG treatment (5 mmol/L) (n = 18). **(S, T)** Fold changes of percentage **(S)** and MFI **(T)** of IFN-γ expression were calculated as the treatment with additional 2-DG treatment (5 mmol/L) over the treatment without 2-DG in CD160^-^ as well as CD160^+^ NK cells from HIV-infected individuals (n = 18). The mean fluorescence intensity of GLUT1 and IFN-γ in **(C, F, N)** was from total NK cells. And the mean fluorescence intensity of IFN-γ in **(H, P, J, R)** was from CD160^-^ and CD160^+^ NK cells, respectively. The fold change of IFN-γ MFI in panels **(L, T)** was from CD160^-^ and CD160^+^ NK cells, respectively. A Wilcoxon matched-pairs signed-rank test was employed for paired-group comparisons. A Spearman’s rank test was applied for correlation analysis; * P < 0.05; ** P < 0.01; *** P < 0.001; **** P < 0.0001; ns, no significance. Data are represented as means ± SD.

First, we examined GLUT1 expression on total NK cells from HIV-infected individuals and found that both the percentage of cells expressing GLUT1 and MFI were enhanced by additional glucose (**Figures 4A–C**). To further explore the function affected by glucose metabolism, we stimulated PBMCs from HIV-infected individuals with IL-12, 15, 18 overnight, with or without additional glucose or 2-DG (**Figure 4D**). We observed that exogenous glucose improved total NK cell IFN-γ production (**Figures 4E, F** **)**. In addition, CD160^+^ NK subset showed both higher percentage and mean fluorescent intensity of IFN-γ production when glucose was added, while CD160^-^ NK cells only exhibited an increased percentage of IFN-γ positivity (**Figures 4G-J**). Glucose treatment played a particularly important role in CD160^+^ NK-cell function, as demonstrated by higher fold change of IFN-γ mean fluorescent intensity levels, while the change in percentage was not statistically significant (**Figures 4K, L** **)**. These findings prompted us to further test whether 2-DG, a glucose analogue taken up by glucose transporters, which functions as a glycolytic inhibitor, could limit IFN-γ production. As expected, IFN-γ production in total NK cells from HIV-infected individuals was decreased after 2-DG treatment (**Figures 4M, N** **)**, and the same result was also found in both CD160^-^ and CD160^+^ NK subsets (**Figures 4O-R**). More importantly, the fold change in IFN-γ in CD160^+^ NK cells were lower than that in CD160^-^ NK cells following 2-DG treatment (**Figures 4S, T**).

Together, these results indicate that CD160 may have a positive regulatory effect on NK-cell glucose metabolism in HIV-infected individuals.

### CD160 Upregulates NK-Cell Glucose Metabolism Through the AKT/mTOR/s6k Signaling Pathway

**Figure 5:**
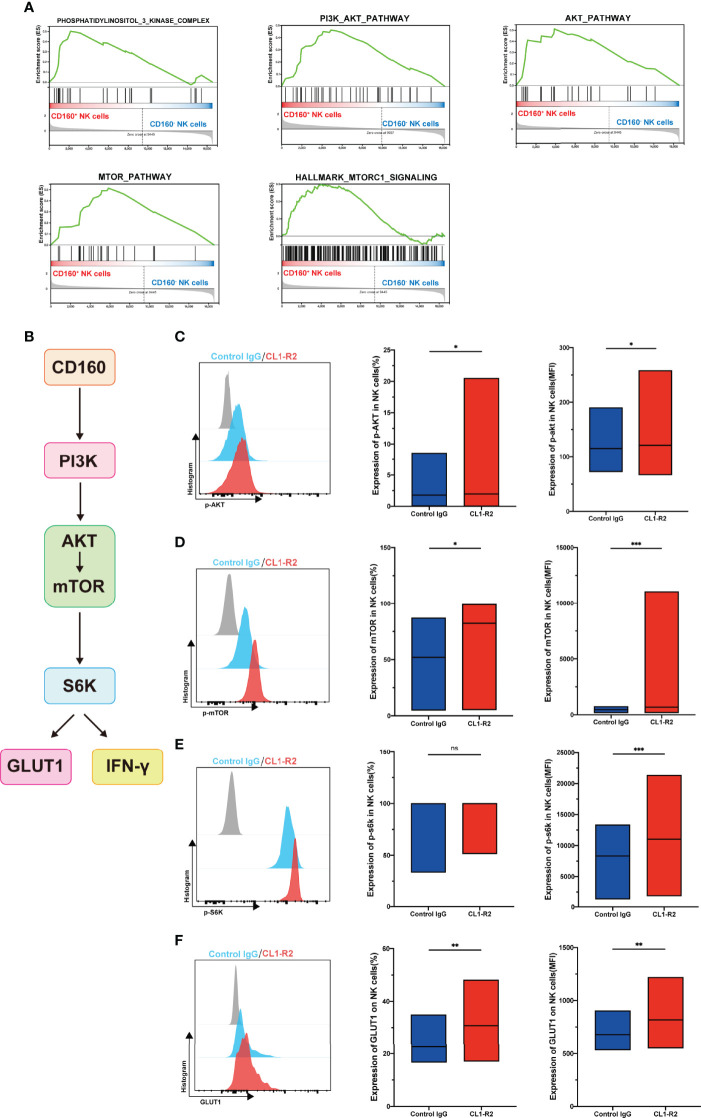
CD160 upregulates NK-cell glucose metabolism through AKT/mTOR/s6k signaling pathway. **(A)** GSEA analysis of RNA-seq data from CD160^-^ or CD160^+^ NK cells. **(B)** Schematic showing the hypothetical signaling pathway triggered by CD160 to regulate NK cell function. **(C–E)** NK cells from HIV-infected individuals were treated with 10 µg/ml CL1-R2 antibody or negative control IgG1 for 30min, then AKT **(C)**, mTOR **(D)**, and s6k **(E)** phosphorylation in total NK cells from HIV-infected individuals were measured by flow cytometry. Representative histogram (left), percentage (middle), and mean fluorescence intensity (right) (n= 28). **(F)** Treated with 10 µg/ml CL1-R2 antibody or negative control IgG1 for 1h, and then stimulated with IL-12 + IL-15 + IL-18 (10, 50 and 100 ng/ml, respectively) for 24h, the expression of GLUT1 on total NK cells from HIV-infected individuals were analyzed (n=11). The mean fluorescence intensity of p-AKT, p-mtOR, p-s6k, and GLUT1 in **(C–F)** was from total NK cells. A Wilcoxon matched-pairs signed-rank test was employed for paired-group comparisons; * P < 0.05; ** P < 0.01; *** P < 0.001; ns, no significance. Data are represented as means ± SD.

Our data demonstrated that CD160 influences NK-cell glucose metabolism and NK-cell function; however, the related signaling pathway was unknown. Our Gene set enrichment analysis (GSEA) showed that, compared with CD160^-^ NK cell subsets, phosphatidylinositol-3-kinase-complex genes were strongly enriched in the CD160^+^ NK cell subsets. GSEA also revealed that data from the CD160^+^ NK cell subsets were correlated with response to PI3K/AKT, AKT and mammalian target of rapamycin (mTOR) signaling, as well as hallmarks of mTORC1 genes (**Figure 5A**).

Since mTOR, a central metabolic regulator, is proposed as an important factor downstream of AKT signaling (33), one of its two distinct complexes, mTORC1, has received substantial research attention. When activated, NK cells undergo a dramatic metabolic reprogramming, including increased glucose uptake and glycolysis, as well as mTORC1 activity, which is widely evaluated using s6k (34), is essential for achievement of this elevated glycolytic state (35). Therefore, we hypothesized that CD160 could enhance NK cell effector functions by upregulating glucose and glycolysis through the PI3K/AKT/mTOR/s6k signaling pathway (**Figure 5B**). To test this hypothesis, we first triggered CD160 on total NK cells from HIV-infected individuals, and found that expression of p-AKT was higher than in isotype control treated cells (**Figure 5C**). Moreover, engagement of CD160 also upregulated phosphorylated mTOR (**Figure 5D**). Consistent with these findings, levels of p70s6k were also elevated, and the mean fluorescence intensity of p70s6k was higher following CD160 activation, indicating enhanced activity of mTORC1 (**Figure 5E**). In addition to CD160 agonist, IL-15 can promote the function of NK cells through the PI3K-AKT-mTOR pathway, and in the presence of PI3K, AKT, or mTOR pathway inhibitors, the proliferation of NK cells and the secretion of IFN-γ stimulated by IL-15 in NK cells were completely abrogated (36). In our study, we used IL-15 to stimulate NK cells and found that the expressions of p-AKT, p-mTOR, and p-s6k were higher in CD160^+^ NK cells (**Figures S6A-F**), and we also found that the expansion of NK cells was higher in CD160^+^ NK cells (**Figures S7A, B**). As shown in **Figure 5F**, levels of GLUT1 on total NK cells from HIV-infected individuals were significantly higher on CL1-R2 treatment than following control IgG treatment, suggesting that CD160 plays a pivotal role in enhancement of GLUT1 expression on NK cells.

### TGF-β Inhibits CD160 Expression on NK Cells

**Figure 6:**
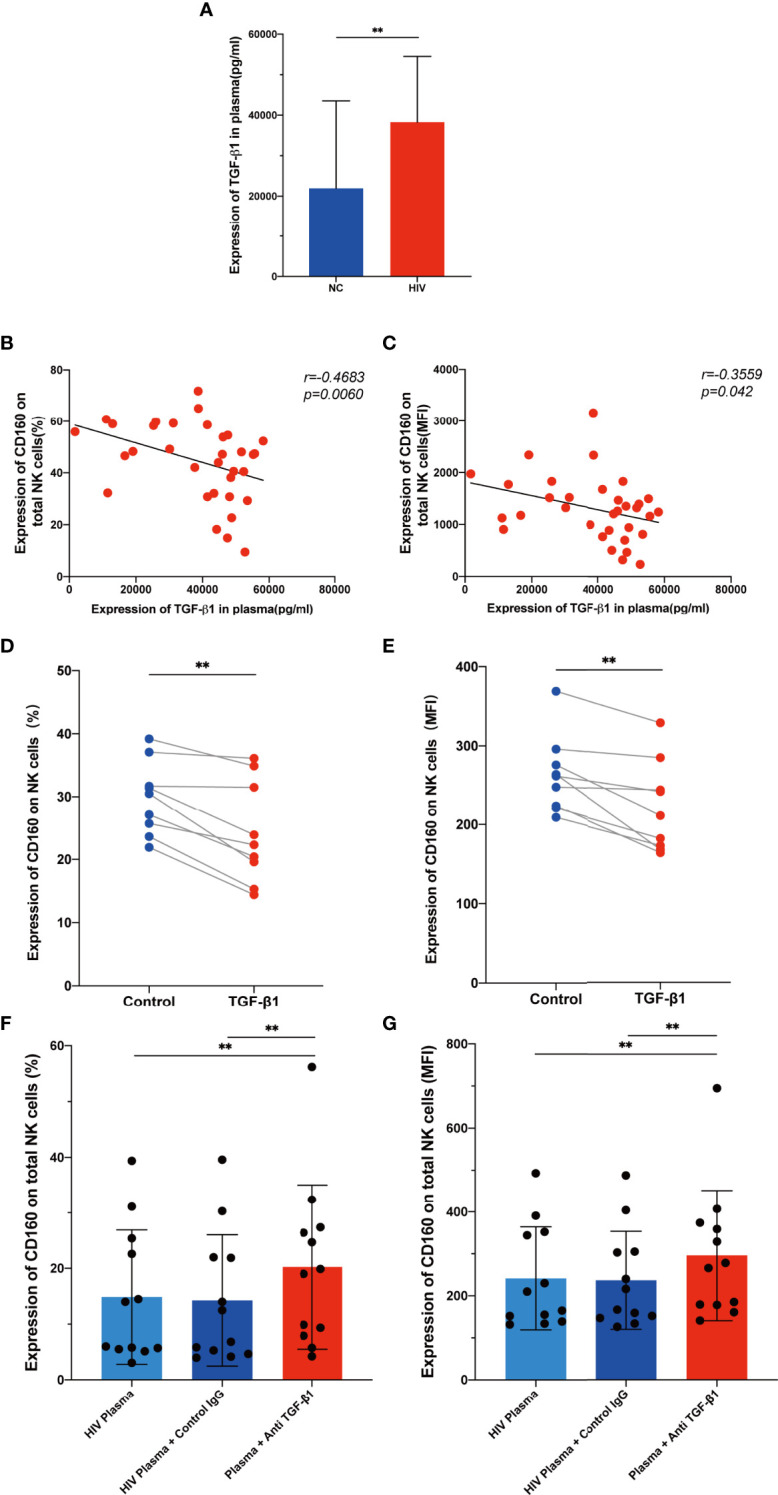
TGF-β inhibits CD160 expression on NK cells. **(A)** Levels of TGF-β1 in plasma from HIV-infected individuals (n = 39) and HIV-negative controls (n = 21). **(B, C)** Correlations between levels of TGF-β1 and the percentages **(B)** or MFI **(C)** of CD160 on total NK cells from HIV-infected individuals (n = 33). **(D, E)** Cultured with medium alone, or with TGF-β1 (10 ng/ml). Percentages **(D)**, and MFI **(E)** of CD160 on total NK cells from HIV-infected individuals were analyzed by flow cytometry (n=9). **(F, G)** PBMCs cultured with HIV-infected individual plasma alone (left), cultured with HIV-infected individual plasma and control IgG (middle), or cultured with HIV-infected individual plasma and anti-human TGF-β1-neutralizing antibody (right), were analyzed by flow cytometry to detect the percentage **(F)** and MFI **(G)** of CD160 on total NK cells from HIV-infected individuals (n = 12). The mean fluorescence intensity of CD160 in **(C, E, G)** was from total NK cells. A Wilcoxon matched-pairs signed-rank test was employed for paired-group comparisons. A Spearman’s rank test was used for correlation analysis; ** P < 0.01. Data are represented as means ± SD.

As a multipotent immunosuppressive cytokine, transforming growth factor beta (TGF-β) is systemically induced during acute HIV infection, and levels are sustained throughout infection (37). We hypothesized that TGF-β may inhibit CD160 expression on total NK cells; therefore, we assessed plasma TGF-β levels in the HIV-infected group and NC group, and found that levels were higher during HIV infection (**Figure 6A**). Interestingly, TGF-β1 plasma levels were negatively correlated with the mean fluorescence intensity and percentage of CD160 on total NK cells (**Figures 6B, C** **)**. To determine whether TGF-β is the reason for the altered CD160 expression, we preincubated NK cells from HIV-infected individuals with 10 ng/ml TGF-β1 for 24h *in vitro*, and observed a significant downregulation CD160 on NK cells, in terms of both percentage and MFI (**Figures 6D, E** **)**. Moreover, we cultured NK cells with HIV-infected individual plasma alone, HIV-infected individual plasma and control IgG, or HIV-infected individual plasma and anti-human TGF-β1-neutralizing antibody, and it was found that anti-TGF-β1 Ab treatment partially restored CD160 expression on total NK cells (**Figures 6F, G** **)**. According to these data, we infer that, higher TGF-β may be a major factor in causing reduced CD160 expression on total NK cells in HIV-infected individuals.

## Discussion

In this article, we found that CD160 expression on total NK cells was significantly lower in HIV-infected individuals and negatively correlated with HIV disease progression. According to our findings, CD160^+^ NK cells also exhibited increasing levels of CD69 and Ki67, as well as producing more TNF-α and IFN-γ, and CD160 expression was strongly associated with Ki67, TNF-α, and IFN-γ production, revealing that CD160 may improve NK cell activation, proliferation, and cytokine production. NK cells from CD160-deficient mice produced reduced levels of IFN-γ and TNF-α, and formed larger tumors than wildtype controls (20–38). We also found that expression of CD160 on total NK cells was positively and negatively correlated with CD4^+^ T cell counts and HIV viral load, respectively, possibly because cells that do not express CD160 have diminished effector functions, leading to an increase in HIV viral load and a decrease in CD4^+^ T cells. When CD160-deficient and wildtype mice were infected with lymphocytic choriomeningitis virus, the viral burden in the plasma, spleen, liver, and kidneys was considerably higher in the CD160-deficient group (29), supporting our results that CD160 plays an essential role in NK cell function.

Metabolism is critical in the activation and specialized functions of immune cells (39), and can alter NK cell destiny and function (40). Given that NK cells strongly rely on glucose metabolism to exert their functions and CD160 plays a vital part in regulating effector functions, we hypothesized that there may be a connection between NK-cell glucose metabolism and CD160 expression on total NK cells in HIV-infected individuals. We found that GLUT1 expression on total NK cells was significantly downregulated in HIV infected individuals; however, the percentages of other nutrient transporters, including CD36, CD71 and CD98 were comparable between the HIV-infected and NC groups. We also found the upregulation of GLUT1 expression and glucose uptake in CD160^+^ NK cells relative to CD160^-^ NK cells from HIV-infected individuals. Moreover, CD160 expression on total NK cells was positively correlated with that of GLUT1 and glucose uptake, indicating that the CD160 may play a key role in glucose metabolism. Furthermore, we observed that IFN-γ production was enhanced by exogenous glucose, consistent with a previous report that increased glycolysis in human NK cells is essential for maximum IFN- responses (41). Interestingly, our data show, for the first time, that fold change of IFN-γ production increased significantly in CD160^+^ NK cells relative to that in CD160^-^ NK cells from HIV-infected individuals on addition of exogenous glucose. Furthermore, the fold change of IFN-γ was significantly decreased in CD160^+^ NK cells when glycolysis was inhibited by 2-DG treatment, demonstrating that CD160 may positively regulate NK cell function *via* glucose metabolism in HIV-infected individuals.

In our study, GSEA showed that genes involved in the PI3K, AKT, and mTOR signaling pathway were enriched in CD160^+^ NK cells, and targeting CD160 signaling using an agonistic mAb, enhanced the phosphorylation of mTOR, AKT and s6k, demonstrating that CD160 regulates NK-cell functions *via* glucose metabolism through the AKT/mTOR/s6k signaling pathway. The co-signaling receptor, PD-1, is reported to inhibit PI3K and AKT phosphorylation, thereby downregulating glucose metabolism (42). In a previous study of gastric cancer, AKT/mTOR/s6k signaling was found to be related to T-cell glucose metabolism (43). As a primary energy sensor, mTOR promotes glycolytic metabolism and integrates signals for nutrition availability, growth, and activation (44). Raymond et al. demonstrated that IFN-γ production in mouse activated NK cells is dependent on activation of mTOR (35). Neethi Nandagopal et al. have demonstrated that in the presence of PI3K, AKT, or mTOR inhibitors, the proliferation of NK cells and the secretion of IFN-γ by NK cells were completely abrogated (36). In addition, Julia A. Wagner et al. also demonstrated that PI3K inhibitor could down regulate the production of IFN-γ in NK cells (45). Moreover, Liang et al. found that mTORC1 could enhance GLUT1 expression on CD8^+^ T cells cocultured with IL-33 (46). We also found that triggering CD160 could significantly enhance GLUT1 levels on NK cells, further indicating a leading role for CD160 in improving glucose metabolism.

Little is known about the mechanism involved in downregulation of CD160 in HIV-infected individuals. TGF-β1 is systemically elevated as early as 1–4 days after viremic dissemination begins in acute HIV-1 infection and remains raised throughout infection (37). TGF-β1 is an immunosuppressive cytokine that negatively regulates IFN-γ production and expression of the activating receptors NKG2D and NKp30, reducing NK cell cytotoxicity and affecting antitumor activity (47). TGF-β antagonizes pro-inflammatory cytokines effects, down-regulating T-bet and IFN-γ *via* SMAD3. Consistently, SMAD3-deficient mouse NK cells produce more IFN-γ. To determine the possible reason for the reduction of CD160 on total NK cells from HIV-infected individuals, we evaluated plasma TGF-β1 levels and found that they were elevated in the HIV-infected group. In addition, we observed a negative correlation between TGF-β1 level and CD160 expression on total NK cells. Further, TGF-β1 treatment greatly decreased CD160 expression on total NK cells cultured *in vitro*. The mechanism by which TGF-β1 downregulates CD160 is unknown. A recent study demonstrated that TGF-β1 can up-regulate human microRNA-1245 expression, thereby downregulating NKG2D expression on NK cells (48). TGF-β1 may induce the production of specific miRNAs which interfere with CD160 transcription, resulting in reduced CD160 expression.

**Figure 7:**
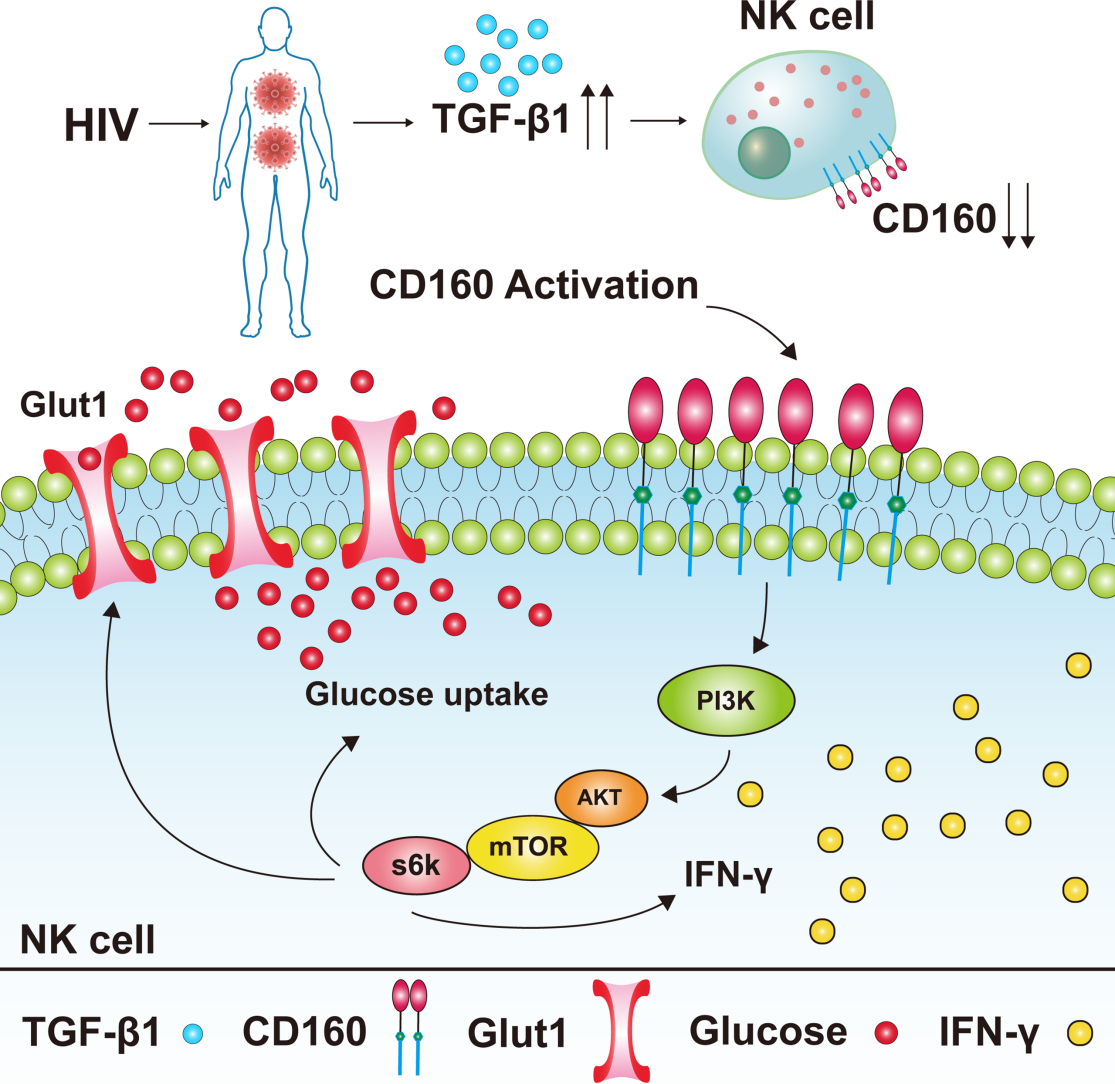
Model of the expression level of CD160 on total NK cells in HIV-infected individuals and its underlying mechanism for NK cell functions. CD160 expression was reduced on total NK cells from HIV-infected individuals, the reason for the reduction of CD160 on total NK cells may be due to the higher plasma levels of TGF-β1 in HIV-infected individuals. The activation of CD160 can enhance GLUT1 expression and the glucose uptake of NK cells in HIV-infected individuals, and finally promotes NK cell functions through the PI3K/AKT/mTOR/s6k signaling pathway.

In summary, our results suggest that CD160 expression is significantly decreased on peripheral total NK cells from HIV-infected individuals, and negatively associated with HIV disease progression. CD160^+^ NK cells from HIV-infected individuals produce more IFN-γ and TNF-α and exhibit a stronger proliferation capability. Furthermore, our data demonstrates that CD160 enhances glucose metabolism *via* the AKT/mTOR/s6k signaling pathway. In addition, we found elevated TGF-β1 levels in plasma from HIV-infected individuals, which can mediate reduction of CD160 (**Figure 7**). Our findings suggest that triggering CD160 may further improve therapeutic effects against HIV infection and provide new insights into immune activation and related pathology.

There is a limitation in our study that should be taken into account when interpreting our results. We demonstrated the effect of CD160 on the glucose metabolism of NK cells in HIV-infected individuals by detecting the expressions of GLUT1 and 2-NBDG on CD160^-/+^ NK cells, and found that the expression of GLUT1 and 2-NBDG on CD160^+^ NK cells were significantly higher than that of CD160^-^ NK cells, indicating that CD160^+^ NK cells have stronger glucose transport and uptake ability. It would be better to conduct the ECAR experiment to fully demonstrate the effect of CD160 on the glucose metabolism of NK cells in HIV-infected individuals. Moreover, in addition to CD160 agonist, IL-15 was used as another stimulus to stimulate NK cells and found that the phosphorylation of AKT-mTOR levels was higher in CD160^+^ NK cells, but this cannot draw the conclusion that CD160^+^ NK responds better than CD160^-^ NK to IL-15 stimulation, and it needs further study.

## Data Availability Statement

The datasets presented in this article are not readily available because of local policies. Requests to access the datasets should be directed to HS, E-mail: hongshang100@hotmail.com, YJ, E-mail: jiangjun55555@163.com.

## Ethics Statement 

The study was reviewed and approved by The Research and Ethics Committee of CMU. Our study was carried out based on the principles enshrined in the Declaration of Helsinki. All patients/participants provided their written informed consent to participant in this study.

## Author Contributions

ZS designed the experiments, analyzed the data, performed the NK cell experiments, and wrote the manuscript. ZS and YL checked the data. ZZ and YF carried out CD4^+^ T cell counts. XH participated in measurement of HIV viral load. QH and HD helped to recruit study participants. HS and YJ devised, and supervised the study and revised the manuscript. All authors contributed to the article and approved the final version.

## Funding

This work was funded by Science Research for the 13th Five-Year Plan of China, to H.S. (2017ZX10201101) and to Y.J. (2018ZX10732101-001-011), and Scientific Research Funding Project of Higher Education in Liaoning province (LJKZ0737).

## Acknowledgments

The authors would like to express gratitude to all the study participants as well as the patient care team at the Red Ribbon outpatient clinic of China Medical University’s First Affiliated Hospital. The writers also appreciate the spelling and grammar checks provided by English-speaking medical editors from the Charlesworth Group (http://charlesworth-group.com).

## Conflict of Interest

The authors declare that the research was conducted in the absence of any commercial or financial relationships that could be construed as a potential conflict of interest.

## Publisher’s Note

All claims expressed in this article are solely those of the authors and do not necessarily represent those of their affiliated organizations, or those of the publisher, the editors and the reviewers. Any product that may be evaluated in this article, or claim that may be made by its manufacturer, is not guaranteed or endorsed by the publisher.
