# Supplementary material for: CD160 Promotes NK Cell Functions by Upregulating Glucose Metabolism and Negatively Correlates With HIV Disease Progression
Source: Front Immunol. 2022 Aug 19;13:854432. doi: 10.3389/fimmu.2022.854432 (PMC9469471; doi:10.3389/fimmu.2022.854432)
Supplement: Supplementary file 1 [file DataSheet_1.pdf]

Figure S1

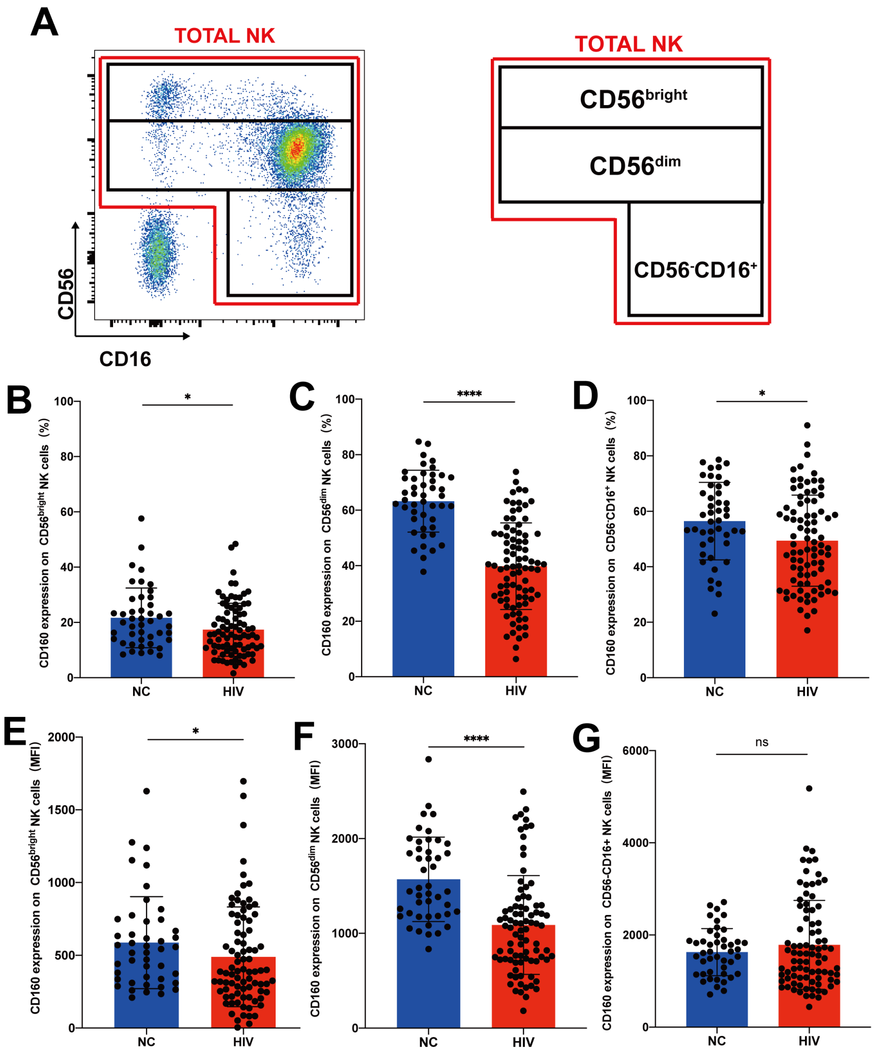

Figure S1. CD160 expression on NK cell subsets.

(A) Schematic diagram of flow cytometry for dividing NK cells into three subsets based on CD56 and/or CD16.

(B—G) Percentage (B—D) and mean fluorescence intensity (MFI) (E—G) of CD160 expression on NK cell subsets from the HIV (n = 85) and NC (n = 45) groups. Data are represented as means ± SD.

Figure S2

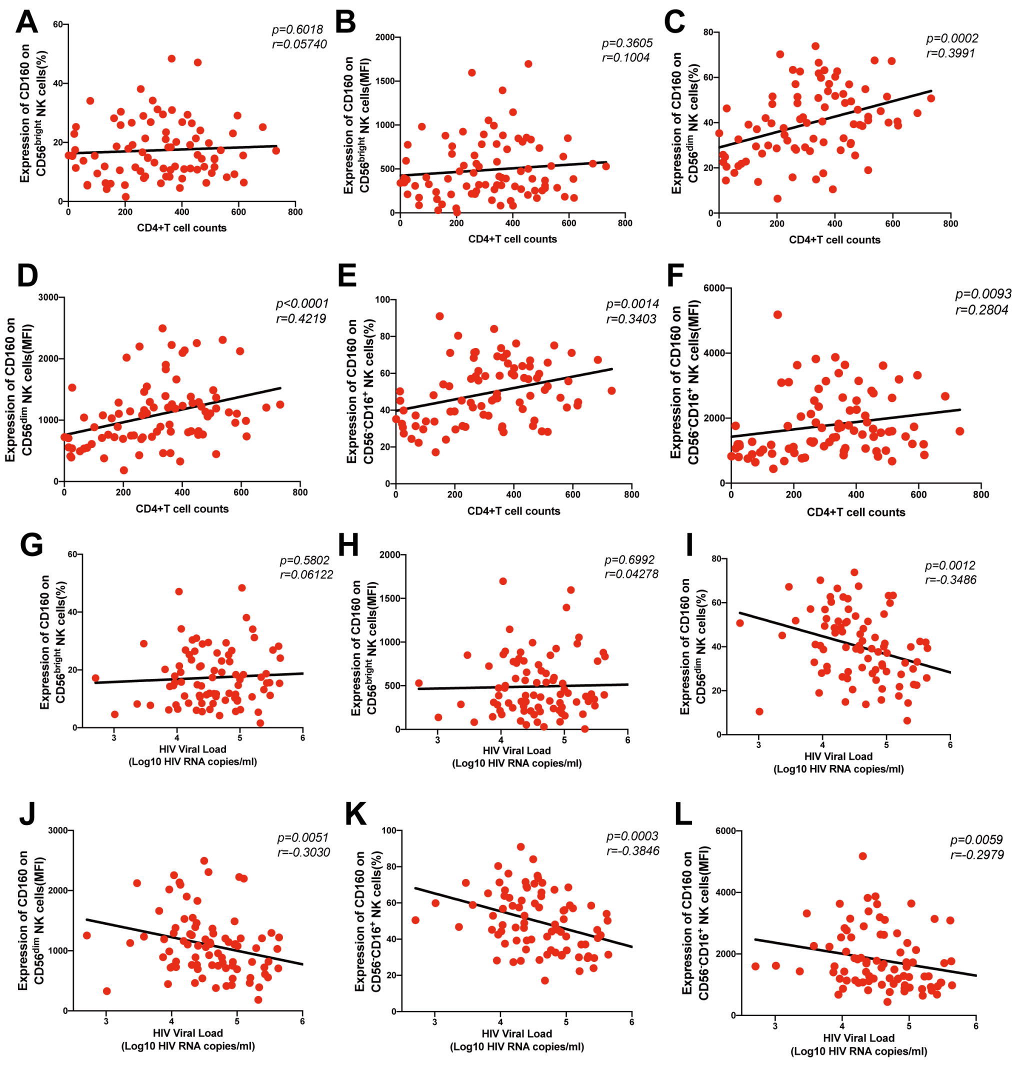

Figure S2. Relationships between CD160 expression on NK cell subsets and the HIV disease progression.

(A—F) Correlation between the CD160 expression on NK cell subsets and the $CD4^{+}$ T cell counts (cells/mm^{3}) (n = 85).

(G—L) Correlation between the CD160 expression on NK cell subsets and HIV plasma viral load (VL) (n = 84).

Figure S3

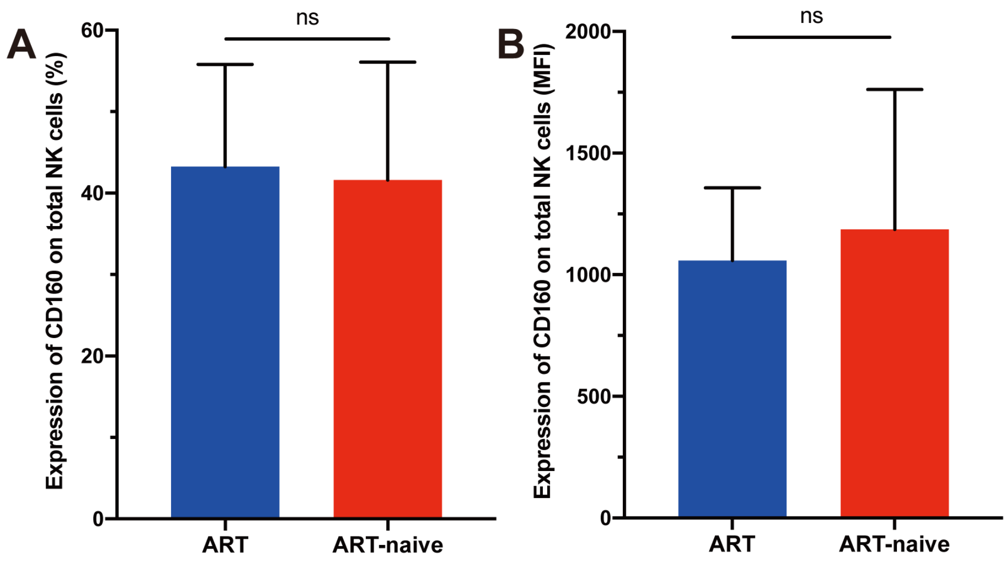

Figure S3. The expression of CD160 on NK cells between ART and ART-naive individuals.

(A) The percentage of CD160 on NK cells between ART and ART-naive individuals (ART: n = 13; ART-naive: n = 85).

(B) The MFI of CD160 on NK cells between ART and ART-naive individuals (ART: n = 13; ART-naive: n = 85).

Data are represented as means ± SD.

Figure S4

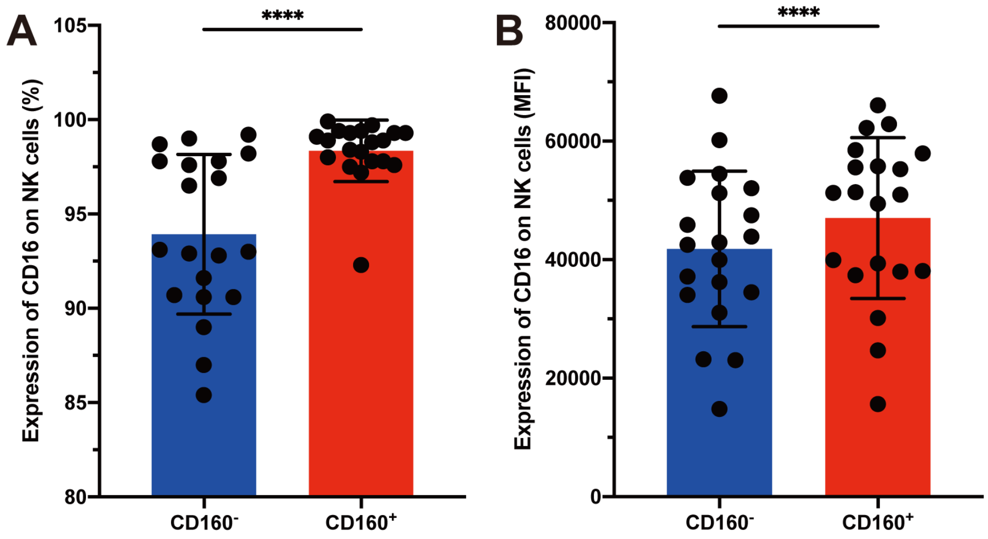

Figure S4. The expression of CD16 between CD160$^-$ and CD160$^+$ NK cells in HIV-infected individuals.

(A) The percentage of CD16 on CD160$^-$ and CD160$^+$ NK cells in HIV-infected individuals (n = 20).

(B) The MFI of CD16 on CD160$^-$ and CD160$^+$ NK cells in HIV-infected individuals (n = 20).

Data are represented as means ± SD.

Figure S5

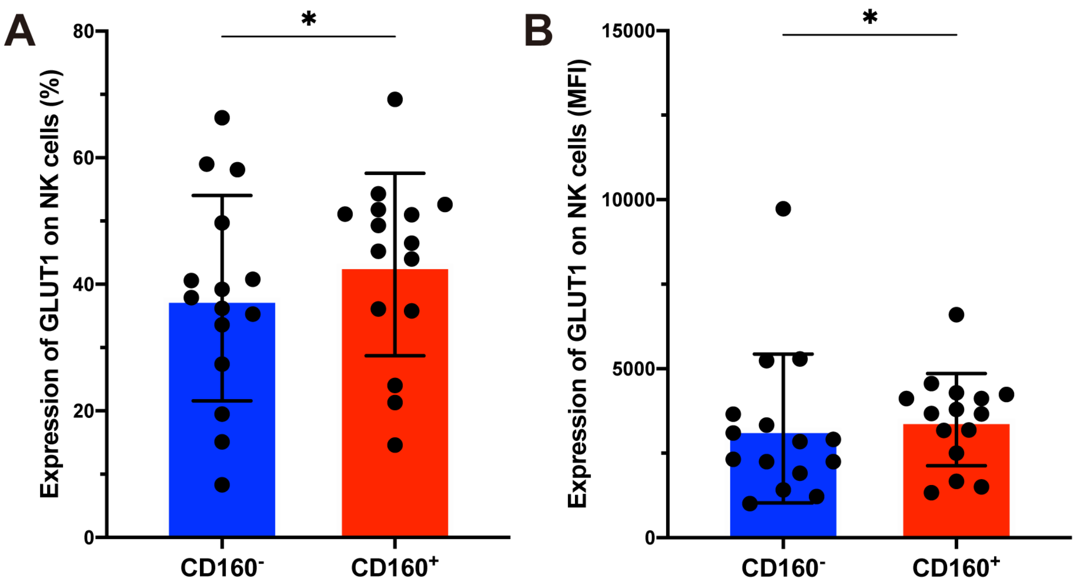

Figure S5. GLUT1 expression on NK cells between CD160⁻ and CD160⁺ NK cells in negative controls.

(A) The percentage of GLUT1 on CD160$^-$ and CD160$^+$ NK cells in negative controls (n = 15).

(B) The MFI of GLUT1 on CD160$^-$ and CD160$^+$ NK cells in negative controls (n = 15).

Data are represented as means ± SD.

Figure S6

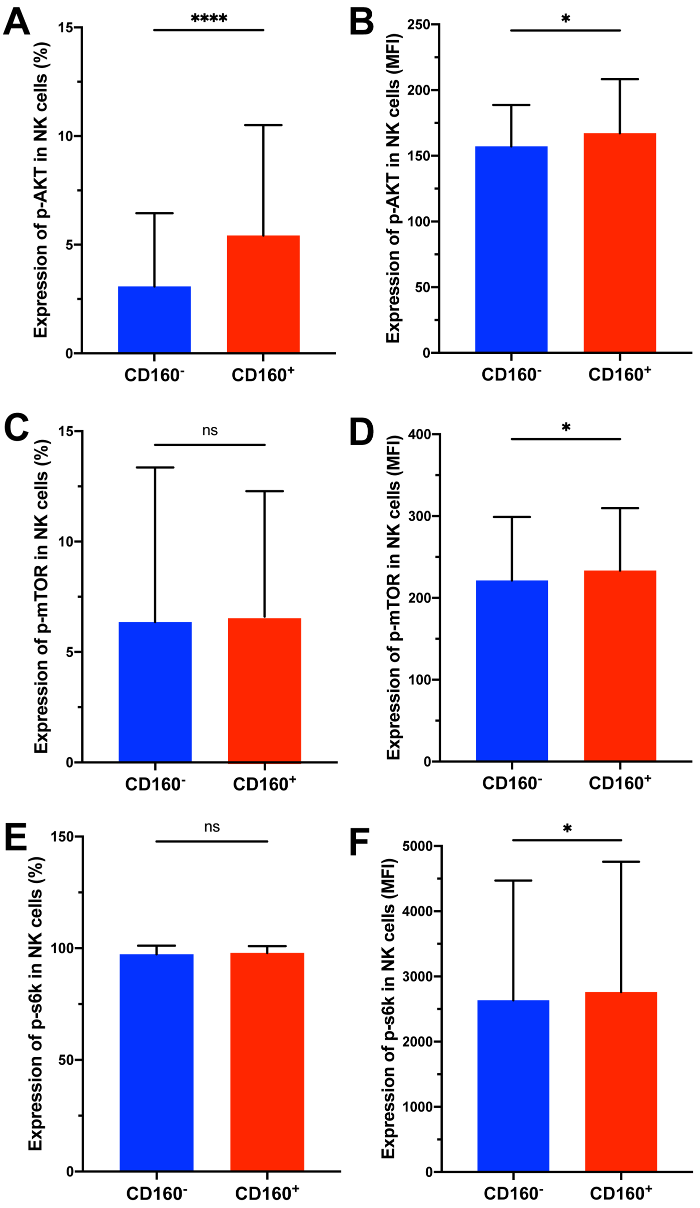

Figure S6. Expression of p-AKT, p-mTOR and p-s6k between CD160$^-$ and CD160$^+$ NK cells.

(A) The percentage of p-AKT between CD160$^-$ and CD160$^+$ NK cells (n = 17).

(B) The MFI of p-AKT between CD160$^-$ and CD160$^+$ NK cells (n = 17).

(C) The percentage of p-mTOR between CD160$^-$ and CD160$^+$ NK cells (n = 14).

(D) The MFI of p-mTOR between CD160$^-$ and CD160$^+$ NK cells (n = 14).

(E) The percentage of p-s6k between CD160$^-$ and CD160$^+$ NK cells (n = 13).

(F) The MFI of p-s6k between CD160$^-$ and CD160$^+$ NK cells (n = 13).

Data are represented as means ± SD.

Figure S7

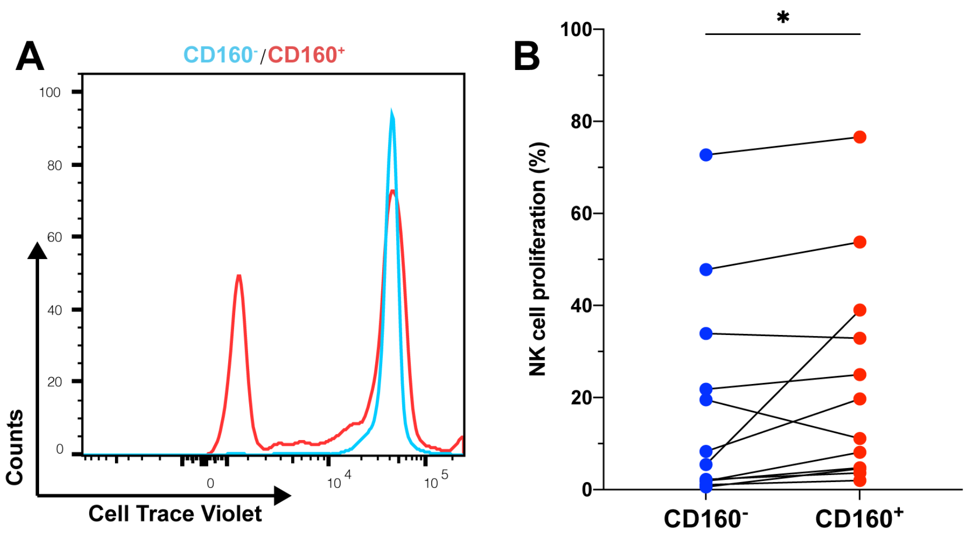

Figure S7. Proliferation of CD160⁻ and CD160⁺ NK cells stimulated by IL-15.

(A) Representative histograms showing the proliferation of CD160⁻ and CD160⁺ NK cells stimulated by IL-15 for 24 hours.

(B) Proliferation between CD160⁻ and CD160⁺ NK cells (n = 12).
